# Establishment of a Community Care Center for Isolation and Management of Ebola Patients — Bomi County, Liberia, October 2014

**Published:** 2014-11-07

**Authors:** Gorbee Logan, Neil M. Vora, Tolbert G. Nyensuah, Alex Gasasira, Joshua Mott, Henry Walke, Frank Mahoney, Richard Luce, Brendan Flannery

**Affiliations:** 1 Bomi County Community Health Department, Tubmanburg, Liberia; 2 Division of Global Health Protection, Center for Global Health, CDC; 3 Ministry of Health and Social Welfare, Monrovia, Liberia; 4 Immunization, Vaccines, and Emergencies Program, Regional Office for Africa, World Health Organization; 5 Influenza Division, National Center for Immunizations and Respiratory Diseases, CDC; 6 Division of High-Consequence Pathogens and Pathology, National Center for Emerging and Zoonotic Infectious Diseases, CDC; 7 Global Immunizations Division, Center for Global Health, CDC

As of October 29, 2014, a total of 6,454 Ebola virus disease (Ebola) cases had been reported in Liberia by the Liberian Ministry of Health and Social Welfare, with 2,609 deaths (1). Although the national strategy for combating the ongoing Ebola epidemic calls for construction of Ebola treatment units (ETUs) in all 15 counties of Liberia, only a limited number are operational, and most of these are within Montserrado County. ETUs are intended to improve medical care delivery to persons whose illnesses meet Ebola case definitions (2), while also allowing for the safe isolation of patients to break chains of transmission in the community. Until additional ETUs are constructed, the Ministry of Health and Social Welfare is supporting development of community care centers (CCCs) for isolation of patients who are awaiting Ebola diagnostic test results and for provision of basic care (e.g., oral rehydration salts solutions) to patients confirmed to have Ebola who are awaiting transfer to ETUs. CCCs often have less bed capacity than ETUs and are frequently placed in areas not served by ETUs; if built rapidly enough and in sufficient quantity, CCCs will allow Ebola-related health measures to reach a larger proportion of the population. Staffing requirements for CCCs are frequently lower than for ETUs because CCCs are often designed such that basic patient needs such as food are provided for by friends and family of patients rather than by CCC staff. (It is customary in Liberia for friends and family to provide food for hospitalized patients.) Creation of CCCs in Liberia has been led by county health officials and nongovernmental organizations, and this local, community-based approach is intended to destigmatize Ebola, to encourage persons with illness to seek care rather than remain at home, and to facilitate contact tracing of exposed family members. This report describes one Liberian county’s approach to establishing a CCC.

In March 2014, the Bomi County Community Health Department (BCCHD) built an isolation ward for Ebola patients adjacent to the county’s single hospital after receiving news of the first Ebola case in Liberia (Figure). Because Bomi County (population: 84,000) borders Montserrado County (3), this 12-bed isolation ward was designed as part of a contingency plan in case patients in Bomi County could not be transferred to an ETU in Montserrado County. On June 19, the first Ebola case was reported in Bomi County in a man aged 40 years who was immediately taken to an ETU in Montserrado County. An additional 12 patients whose illnesses met case definitions for suspected or probable Ebola were identified in July 2014, 11 of whom were transferred to Montserrado County and one of whom died before transfer. Four of these 12 Ebola cases occurred among health care workers who had attended the same funeral, and mounting concerns about infection control prompted closure of the county hospital and all 23 clinics in Bomi County by late July. When the facilities reopened nearly 1 month later, ETUs in Montserrado County were no longer accepting transfers; on August 18, 2014, the Bomi County isolation ward therefore admitted its first patient with suspected Ebola. As the isolation ward’s census grew, patients whose illnesses met case definitions for suspected, probable, and confirmed Ebola were assigned to different areas of the ward that were separated by incomplete partitions.

On October 9, 2014, a second newly constructed 15-bed ward was opened adjacent to the original isolation ward. Both wards are staffed by BCCHD health care workers 24 hours per day and by trained Ebola survivors from the community. BCCHD has also provided boarding space for relatives of admitted patients who do not live near the hospital to facilitate patient visits and provision of food and support for patients. Additional assistance with operations (e.g., performing safe burials) and supplies (e.g., personal protective equipment) have been provided by local civil society and concerned private citizens; the pivotal role played by various segments of the community led to these two complementary wards being labeled as a CCC.

Infection control is a major concern within the CCC for patients, health care workers, and the lay community. For example, patients suspected of having Ebola but who do not actually have Ebola will occasionally be admitted to the CCC. These patients might remain within the CCC for days before receiving their diagnostic test results confirming their Ebola-free status, during which time they are at risk for an Ebola virus exposure within the CCC itself. All patients discharged from the CCC after testing negative for Ebola are therefore monitored for Ebola symptoms daily for 21 days by trained BCCHD personnel, regardless of whether the patients are discharged to home or to the hospital for additional non-Ebola care. To reduce the risk for health care–associated Ebola virus infections within the CCC, BCCHD separates patients between the two wards according to their risk for transmitting Ebola virus. The first ward is exclusively for patients with confirmed Ebola and for patients with severe diarrhea, vomiting, or bleeding who have not been confirmed to have Ebola but who would be highly infectious if they had Ebola. The second ward is designated for patients not confirmed to have Ebola and who do not have severe diarrhea, vomiting, or bleeding. Materials, patients, and staff move in one direction, from lower-risk areas (second ward) to higher-risk areas (first ward). For example, if a patient in the second ward experiences severe diarrhea, vomiting, or bleeding, or if laboratory testing confirms that the patient has Ebola, then the patient is moved to the first ward. Given the risks of working in the CCC, BCCHD staff and Ebola survivors undergo infection control training with personal protective equipment before being allowed to enter the CCC. Members of the community are not permitted to come into direct contact with patients and rely on staff to deliver goods to patients.

Since June 19, 2014, Bomi County has reported 72 confirmed, 43 probable, and 62 suspected Ebola cases (1). BCCHD established its own CCC in response to this growing case load and because ETUs in Montserrado County were not accepting patient transfers; this CCC now serves as a regional referral center for neighboring counties. An ETU supported by the U.S. Department of Defense is currently under construction in Bomi County, and BCCHD and community leaders are discussing the possibility of building a second CCC in a more remote region of Bomi County where a cluster of cases was recently identified. Once the ETU is functional, the Bomi County CCCs will be disinfected and be used as a holding place for persons with high-risk Ebola virus exposures to allow for close follow-up and response in case any of these persons develop Ebola.

Although CCCs are being used as an interim solution to the current shortage of functioning ETUs, there is an urgent need to monitor and evaluate this strategy, including whether CCCs have an impact on Ebola virus transmission within the community. To promote consistency in layout, infection control, and clinical management within CCCs, the Ministry of Health and Social Welfare and international partners have developed operational guidelines for CCCs. Trainings are underway to address shortages of staff who are capable of working safely in CCCs to reduce the risk for health care–associated Ebola virus infections. Counties will need ongoing technical assistance to improve triage processes at all county health care facilities so that patients presenting for care whose illnesses meet suspected or probable Ebola case definitions are correctly identified, while also minimizing ETU referrals for patients whose illnesses do not meet suspected or probable Ebola case definitions. Given projections that this outbreak will continue for months (4), there is a need to develop decentralized capacity to manage patients with Ebola, and CCCs are a possible means for quickly developing a local infrastructure for isolation and care of ill persons.

## Figures and Tables

**FIGURE:**
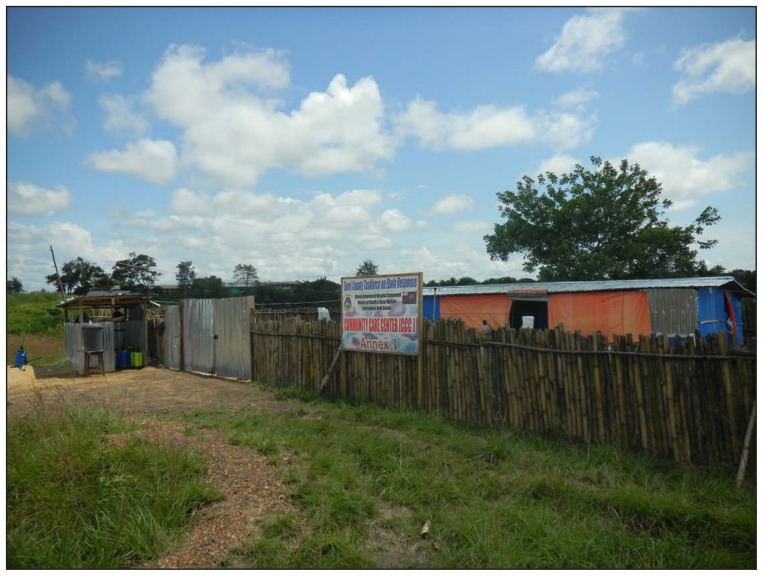
Bomi County community care center, Liberia* Photo/Neil M. Vora * The structure shown here was built by the Bomi County Community Health Department as an isolation ward for Ebola patients in March 2014 after receiving news of the first Ebola cases in Liberia. A second ward was opened adjacent to this one in September 2014, and together these wards function as a community care center. The ward shown here is exclusively for patients with confirmed Ebola and for patients with severe diarrhea, vomiting, or bleeding who have not been confirmed to have Ebola but who would be highly infectious if they had Ebola.
